# Role of Specific Quorum-Sensing Signals in the Regulation of Exopolysaccharide II Production within *Sinorhizobium meliloti* Spreading Colonies

**DOI:** 10.1371/journal.pone.0042611

**Published:** 2012-08-13

**Authors:** Mengsheng Gao, Andrew Coggin, Kruti Yagnik, Max Teplitski

**Affiliations:** Soil and Water Science Department, University of Florida/Institute of Food and Agricultural Sciences, Gainesville, Florida, United States of America; The Scripps Research Institute and Sorrento Therapeutics, Inc., United States of America

## Abstract

**Background:**

Quorum sensing (QS) in *Sinorhizobium meliloti* involves at least half a dozen different *N*-acyl homoserine lactone (AHL) signals. These signals are produced by SinI, the sole AHL synthase in *S. meliloti* Rm8530. The *sinI* gene is regulated by two LuxR-type transcriptional regulators, SinR and ExpR. Mutations in *sinI*, *sinR* and *expR* abolish the production of exopolysaccharide II (EPS II).

**Methodology/Principal Findings:**

This study investigated a new type of coordinated surface spreading of Rm8530 that can be categorized as swarming. Motility assays on semi-solid surfaces revealed that both flagella and EPS II are required for this type of motility. The production of EPS II depends on AHLs produced by SinI. Of these AHLs, only C_16:1_- and 3-oxo-C_16:1_-homoserine lactones (HSLs) stimulated swarming in an ExpR-dependent manner. These two AHLs induced the strongest response in the *wggR* reporter fusions. WggR is a positive regulator of the EPS II biosynthesis gene expression. The levels of the *wggR* activation correlated with the extent of swarming. Furthermore, swarming of *S. meliloti* required the presence of the high molecular weight (HMW) fraction of EPS II. Within swarming colonies, a recombinase-based RIVET reporter in the *wggR* gene was resolved in 30% of the cells, indicating an enhanced regulation of EPS II production in the subpopulation of cells, which was sufficient to support swarming of the entire colony.

**Conclusions/Significance:**

Swarming behavior of *S. meliloti* Rm8530 on semi-solid surfaces is found to be dependent on the functional QS regulatory cascades. Even though multiple AHL signals are produced by the bacterium, only two AHLs species, C_16:1_- and 3-oxo-C_16:1_-HSLs, affected swarming by up-regulating the expression of *wggR*. While EPS II is produced by Rm8530 as high and low molecular weight fractions, only the HMW EPS II facilitated initial stages of swarming, thus, suggesting a function for this polymer.

## Introduction

*S. meliloti* is a soil α-proteobacterium, best known for its ability to establish nitrogen-fixing symbioses with plant hosts belonging to the genera *Medicago*, *Melilotus* and *Trigonella*. Signaling and regulatory events that take place during the early stages of the symbioses are studied and some of these pathways are well defined [1]–[3]. Less studied are the behaviors of this bacterium outside the hosts that influence the symbioses, such as bacterial quorum-sensing signaling in the rhizosphere [4], biofilm formation [5], [6] and the movement of the rhizobium on surfaces [7]–[9].

Bacteria use various types of motility to relocate their populations on surfaces in search for a more suitable environmental niche [10]. Types of surface motility include swarming, sliding, gliding, and twitching [11]. It is thought that motility in rhizobia is critical for the establishment of the symbiosis under natural conditions [8] because it helps the bacteria to gain better access to nutrients, expand into new inches and colonize hosts.

Swarming motility is a multicellular bacterial movement across a surface. It is driven by rotating flagella and coupled to the production of a mucoid layer that facilitates the movement [11], [12]. The latter serves as surfactants to reduce tension between the substrate and the bacterial cells at the swarming front [13] or as wetting agents to extract water from the surroundings [11], [12]. Surfactants and wetting agents can be costly to synthesize, but once released, benefit other cells within the range, thus leading to their characterization as “public goods” [14]. The benefits (as well as costs) and mechanisms of such cooperative behaviors are a subject of research [15]–[17]. The productions of some of those public goods are controlled by quorum sensing (QS) systems [18], [19]. Beside function as QS signals, AHLs with long *N*-acyl chains also function as surfactants in *Rhizobium etli* [20].

*S. meliloti* strain Rm8530 uses the AHL synthase SinI to produce at lease seven AHL molecules. They are C_12_-HSL, C_14_-HSL, 3-oxo-C_14_-HSL, C_16_-HSL, 3-oxo-C_16:1_-HSL, C_16:1_-HSL, C_18_-HSL [21], [22]. At least two LuxR type transcriptional regulators, SinR and ExpR, regulate the expression of *sinI* [23]–[25]. In the presence of SinI AHLs, ExpR controls the accumulation of dozens of transcripts including those encoded by the EPS II gene cluster [24], [26], [27].

EPS II, a galactoglucan polymer, is one of the two symbiotically important exopolysaccharides produced by *S. meliloti* Rm8530 [23], [28], [29]. EPS II is secreted in two fractions, high and low molecular weights. A low molecular weight (LMW) EPS II fraction consists of 15–20 disaccharide subunits, it allows the rhizobial nodule invasion in *Medicago sativa* [30], and it is also critical for the biofilm formation and autoaggregation under laboratory conditions [6], [31]. The function for the high molecular weight (HMW) EPS II fraction has remained elusive.

The EPS II gene cluster contains 22 genes. It is organized into *wge* (also called *expE*), *wga* (*expA*), *wgd* (*expD*), *wggR* (*expG*) and *wgcA* (*expC*) operons [23], [32]. WgcA is critical for the initiation of EPS II biosynthesis. Proteins encoded by *wge* (*expE*), *wga* (*expA*), and *wgd* (*expD*) operons are responsible for the polymerization of EPS II [33]. WggR, a member of MarR family of regulators, activates *wga*, *wgd*, *wggR*, *wgcA* and *wgeA* operons by interacting with the conserved palindrome motifs in the target promoter regions [34], [35]. Disruption of *wggR* prevents the production of EPS II [33]. ExpR stimulates the expressions of *wggR* [24] and other EPS II genes in the presence of SinI AHL and WggR protein [27]. MucR, another regulatory protein, negatively affects the EPS II synthesis by repressing *wgaA*, *wgdA*, and *wggR* genes [8], [36], [37]. Disruption of *mucR* promotes synthesis of the HMW EPS II fraction [30]. In addition, the synthesis of EPS II in *S. meliloti* is also regulated by phosphate starvation [35], [37].

In this study, we first describe the characterization of a flagella- and EPS II-dependent surface swarming behavior of *S. meliloti* Rm8530. We then investgated how AHL signals produced by Rm8530 contribute to the regulation of the bacterial swarming. We found that HMW EPS II is central for the intiation of swarm and that the production of EPS II is controlled by the specific SinI AHLs through stimilating the expression of regulatory gene *wggR*.

## Results

### A structured surface spreading behavior in *S. meliloti* Rm8530

**Figure 1:**
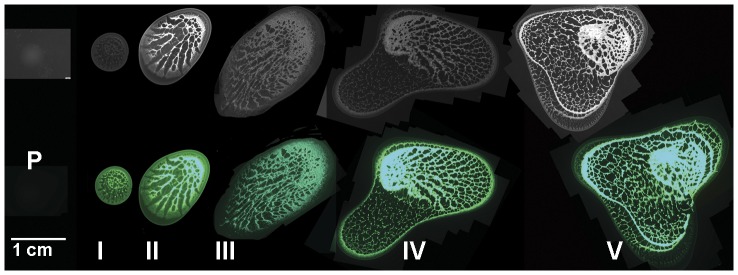
Stages of *S. meliloti* Rm8530 colony spreading. A series of dark-field (top panels) and its corresponding fluorescent (lower panels) images of a *S. meliloti* Rm8530 pDG71 (marked with constitutively expressed *gfp*) swarming colony spreading over a 0.4% agar surface. Stages of spreading that are shown: pre-spreading (stage p, 3 hours after incubation); formation of a Swiss chees-like appearance and initiation of spreading (stage 1, 20 hours after incubation); formation of feather-like patterns and continuation of the colony spreading (stage II, III, 40 h and 3 days after inoculation, respectively); complex patterns and later stages of the colony spreading (stage IV and V, 4 day and 5 days after incubation respectively). Micrographs were taken using a digital camera connected to a dissecting microscope. If a single colony could not be captured as one image, images were assembled in Adobe Photoshop CS, and edges of assembled images were left visible.

As shown in Fig. 1, *S. meliloti* strain Rm8530 formed large mucoid colonies that spread slowly over the surface of very soft agar (0.4%) and developed distinct patterns. Even though *S. meliloti* 8530 bacteria were previously shown to slid on a harder agar medium (0.6%) [8], [9] and spread on regular agar medium (1. 5%) [24], [33], the distinct patterns observed in Fig. 1 were not seen under those conditions [8], [9], [33]. The structured surface spreading colony of Rm8530 was enclosed within an extracellular mucoid matrix and had an entire edge. Spreading was more pronounced when the agar was based on 20-fold-diluted Luria-Bertani (LB) medium.

To better visualize the patterns within the spreading colony of Rm8530, the bacteria were marked with pDG71which contains a constitutively expressed *gfp* gene for green fluorescent protein (GFP) [38]. The spreading colony formation was documented over a five-day period under a microscope and several distinctive stages in the formation of the spreading colony were observed (Fig. 1).

After 10–14 hours of incubation of Rm8530 on the soft agar, an uneven distribution of bacterial cells within the pre-swarming colonies was observed, resulting in many “terraces” and “valleys” or Swiss cheese-like structure in the center of the colonies. A few hours later, colonies appeared “wet” and began to spread. The colonies moved at 0.15–0.3 µm/s (0.5–1 mm/h) between day 1 (I-stage) and day 3 (III-stage) after the inoculation (Fig. 1). The colonies were immersed in and were apparently guided by the almost transparent mucoid matrix. The colony eventually developed a feather-like morphology with pools and channels of slime (Fig. 1). By spreading over the surface, the bacteria multiplied to higher numbers, presumably by gaining access to nutrients. Based on the optical density (OD_600_) measurements, in 3–4 days the total number of cells within the spreading colonies of Rm8530 was estimated to be 2–3 times higher than those within colonies of mutants that were unable to spread (Fig. S1). However, growth rates of these strains in shake cultures were nearly identical.

### Role of flagella in the surface spreading

**Figure 2:**
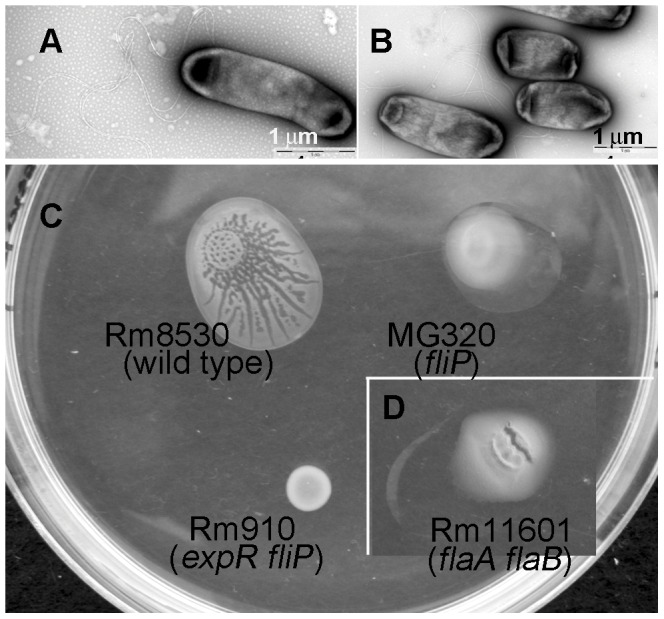
The role of flagella in the surface spreading of *S. meliloti* 8530. **A.** Electron micrographs of a flagellated *S. meliloti* Rm8530 cell from a two-day old spreading colony formed on soft 0.4% agar and flagella-less *S. meliloti* 8530 bacteria from a non-spreading colony formed on hard 1.5% agar (**B**). **C.** Appearance of colonies formed by Rm8530 (wild type), the non-flagellated mutant MG320 (*fliP*) and the EPS II and non-flagellated mutant RmG910 (*expR, fliP*) after two days of spreading on 0.4% agar. **D.** Appearance of a colony formed by a flagellin mutant Rm11601 (*flaA, flaB*) after four days of incubation under similar conditions. Copious amounts of mucoid EPS II are seen on the edges of colonies formed by MG320 and Rm11601.

Because hyper-flagellation is often associated with bacterial swarming [11], [12], over 70 of Rm8530 cells collected from the spreading colonies were examined under a scanning electron microscope (SEM) (Fig. 2A) for the number of flagella associated with cells. No hyper-flagellation was observed. The majority of cells had two to four flagella per cell (Fig. 2A), which is consistent with an earlier report of two to six flagella per swimming *S. meliloti* cell [39]. Cells collected from colonies formed by Rm8530 on hard agar were not associated with flagella (Fig. 2B). The presence of flagella on the cells recovered from the spreading colonies suggested that this type of spreading is distinct from sliding, which is defined as a passive expansion over semi-solid surfaces within a mucoid layer [12].

**Figure 3:**
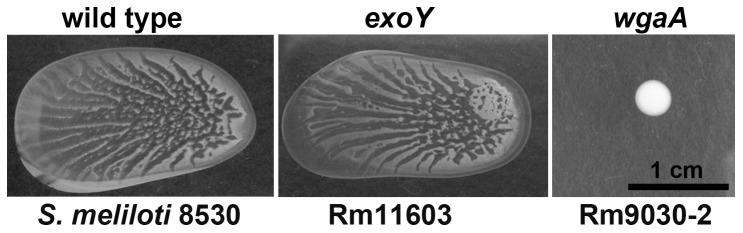
The role of EPS I and EPS II in surface spreading. When *S. meliloti 8530* mutants defective in EPS I (*exoY*, Rm11603) and EPS II (*wgaA*, Rm9030-2) biosynthesis were tested on 0.4% soft agar surfaces, only EPS II-deficient mutant was unable to spread.

To confirm the nature of this type of surface spreading, we tested non-flagellated mutants MG320 (*fliP*) and Rm11601 (*flaA*, *flaB*) [40] for their ability to form structured spreading colonies on soft agar surfaces. Both mutants produced copious amounts of EPS II. However, the MG320 mutant did not form distinct feather-like patterns and did not spread as fast as the wild type (Fig. 2C). Similar results were observed for Rm11601 mutant (Fig. 2D). These observations confirmed that flagella are needed for this type of motility. In Rm910 [41], a mutant strain lacking both *expR* and *fliP*, the surface spreading was completely abolished, indicating that both EPS II and flagella are required for spreading (Fig. 3C). Because both flagella and EPS II are required for this type of motility, it can be characterized as swarming.

### EPS I is not involved in the surface spreading

Because the *S. meliloti* Rm8530 swarming colonies are enclosed within a mucoid matrix, and the bacterium secretes two exopolysaccharides, EPS I and EPS II, experiments were conducted to determine whether or not both EPS I and II are involved in the swarming. Swarming phenotypes of the isogenic EPS I mutant Rm11603 (*exoY*) [40] and Rm9030-2 (*wgaA*) [24] were analyzed (Fig. 3). The *exoY* gene encodes an enzyme that is involved in the initiation of the assembly of repeating units of EPS I [37], [42]. The disruption of *exoY* did not affect swarming, while a mutation in *wgaA* abolished swarming. These results indicate the EPS I has no major function in Rm8530 swarming.

### Role of QS in Rm8530 swarming

The *sinI* and *expR* mutants of *S. meliloti* Rm8530 have been previously shown to be incapable of forming spreading colonies on soft agar (0.3%), implying that QS contributes to Rm8530 swarming [22]. There are at least two possibilities to address the role of QS in the swarming: AHLs may directly facilitate spreading (as reported for *R. etli*) [20], or they may set off a QS regulatory cascade that leads to the expression of the genes involved in the production of the EPS II.

**Figure 4:**
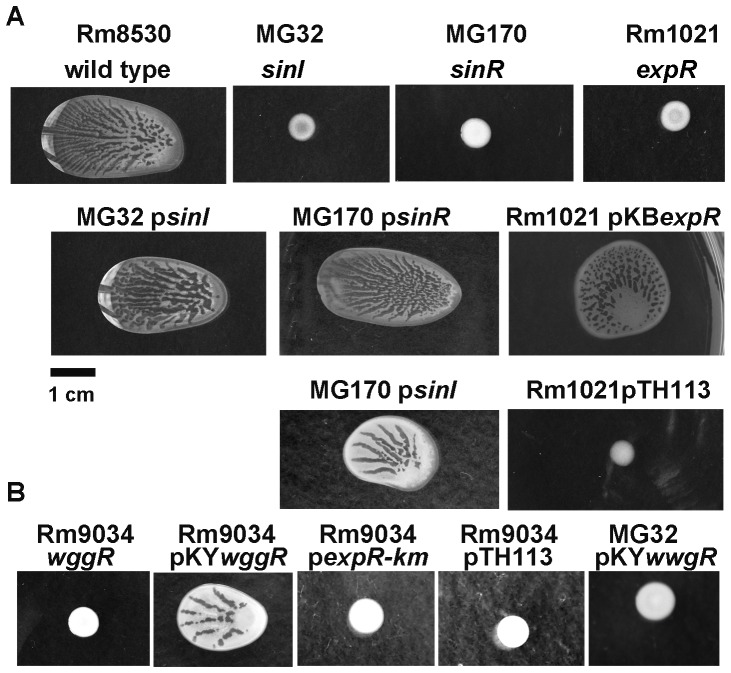
Contributions of Quorum Sensing genes to surface spreading. **A**. Colony spreading phenotypes of Rm8530 (wild type), MG32 (*sinI*), MG170 (*sinR*) and Rm1021 (*expR*) (top row); Complementation and epistasis experiments with the genes of interest supplied in trans: MG32 p*sinI*, MG170 p*sinR*, and Rm1021pKB*expR* (second row); MG170 p*sinI* and Rm1021 pTH113 (carrying an 8.5 kb genomic fragment containing *sinI* and *sinR* genes) (third row) **B**. Colony spreading phenotypes of Rm9034 (*wggR*), Rm9034 pKY*wggR*, Rm9034 p*expRkm*, Rm9034 pTH113, MG32 pKY*wggR*. MG32, MG170, Rm9034 are mutants directly derived from Rm8530. Rm1021 is *expR*-progenitor of *S. meliloti* 8530.

As shown in Fig. 4A (and consistent with previous reports [22], [27]), colonies of the *sinI* mutant MG32, the *sinR* mutant MG170, and the *expR* mutant Rm1021 were dry and did not spread. Complementation of the *sinI* mutant MG32 with p*sinI*, a vector carrying *sinI* gene with *sinI* promoter (downstream and in the same direction as the vector-borne *lac* promoter that is functional in rhizobia [43]) fully restored swarming (Fig. 4A). Complementation of the *sinR* mutant MG170 with p*sinR*, a vector carrying *sinR* gene with the *sinR* promoter, fully restored swarming (Fig. 4A). Because SinR is a known positive transcriptional regulator of *sinI* [44], we further tested the effect of *sinI* (supplied *in trans*) on the behavior of MG170. The introduction of p*sinI* into MG170 partially restored the colony spreading phenotype of the *sinR* mutant (Fig. 4A). This partial restoration of swarming in MG170 by p*sinI* likely reflects the transcription of *sinI* from the plasmid-borne *lac* promoter. These results suggest that the major function of SinR in swarming is restricted to its role in controlling the expression of *sinI*. Complementation of the *expR* mutant Rm1021 with p*expR*, a vector carrying *expR* gene with the *expR* promoter (placed in the same direction and downstream from the vector-borne *lac* promoter), restored swarming. However, the pattern was distinct from that of the wild type (Fig. 4A). The introduction of pTH113 (which carries an 8.5 kb fragment of *S. meliloti* chromosome including *sinRI*) [45] did not override the swarming defect of the *expR* mutation in Rm1021, and the Rm1021 pTH113 strain formed dry colony (Fig. 4A). These results suggest that a functional ExpR is responsible for the perception of the AHLs and/or the regulation of the genes involved in swarm of Rm8530.

To follow up on the hypothesis that ExpR-mediated regulation was central to surface spreading, phenotypes of the genes controlled by ExpR and involved in EPS II biosynthesis were tested. Since WggR activates the expression of EPS II genes and that the expression of *wggR* is stimulated by ExpR [27], we tested the strain Rm9034 [24], a *S. meliloti* 8530 derivative carrying a mutation in *wggR*, for its colony morphology on soft agar. As shown in Fig. 4B, Rm9034 formed a dry colony that did not spread. The introduction of pKY*wggR*, a vector carrying *wggR* regulated by both its native promoter and a *lac* promoter from the vector, restored the swarming phenotype, but the size of the colony was less than that of the wild type Rm8530 (Fig. 4B). This suggests that the timing and the level of *wggR* expression are likely important. The pKY*wggR* plasmid did not restore the swarming defect of the *sinI* mutant (Fig. 4B). The inability of overexpressed *expR* or *sinR* and *sinI* to restore the swarm phenotype of the *wggR* mutant (Fig. 4B) suggests that these gene products contribute little to the swarm phenotype without WggR. The inability of pKY*wggR* to over-ride the *sinI* mutation (Fig. 4B) is consistent with the previously documented [27], [33] direct involvement of ExpR-AHL complexes in the regulation of *wggR* and some other EPS II gene expression.

### Time course of *sinI* and *wggR* expression during Rm8530 swarming

**Figure 5:**
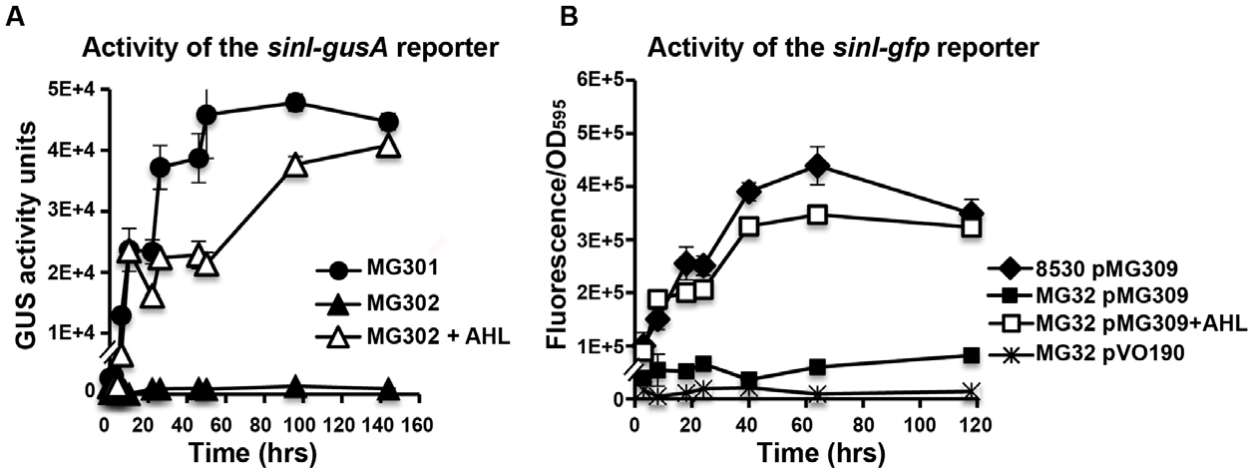
Expression of *sinI*. **A.** Average GUS activity of the chromosomal *sinI-gusA* reporter was measured in the wild type background (MG301, filled circles), *sinI* (MG302, filled triangles), and MG302 with 150 nM of C_16:1_-AHL (open triangles). **B.** Average GFP activity (fluorescence/OD_595_) of the *sinI-gfp* fusion reporter pMG309 in the wild type (filled diamonds), in MG32 (filled squares), and in MG32 with 150 nM of C_16:1_-AHL (open squares). Fluorescence of the plasmid pVO190 (which carries promoterless *gfp*) in MG32 is shown as line with a star. Averages of three technical replications are shown. Error bars present standard deviation. For both assays, bacteria were collected from soft agar surfaces. Plates contained either 150 nM of C_16:1_-AHL (open symbols) or solvent only (methanol) (filled symbols).

To begin to understand the dynamics of the QS regulation in Rm8530 swarming, activities of a chromosomal *gusA* reporter and a plasmid-borne *gfp* reporter, each fused separately with *sinI* and *wggR*, were measured several times during bacterial growth on the surface of 0.4% agar. In the wild type background, the expression of the *sinI-gusA* (MG301, Fig. 5A) increased after five hours of growth within the colony on soft agar (consistent with the late Stage P, before the appearance of patterns within the colony, Fig. 1) and then kept increasing throughout almost the entire course of the swarming. The expression of the same *sinI-gusA* reporter in the *sinI* background remained at low levels (MG302, Fig. 5A). The addition of C_16:1_-HSL, one of several SinI AHLs, added into the soft agar increased the activity of MG302 to nearly wild type levels (Fig. 5A). This confirms that *sinI* is autoregulated within swarming colonies. This conclusion was further supported by testing the activity of pMG309 (a plasmid carrying a *sinI-gfp* fusion) in the wild type strain Rm8530, in the *sinI* mutant MG32, and in the *sinI* mutant grown on the soft agar containing C_16:1_-HSL. The expression-profiles of *sinI-gfp* were similar to those of *sinI-gusA* fusions (fig. 5B compared to 5A).

**Figure 6:**
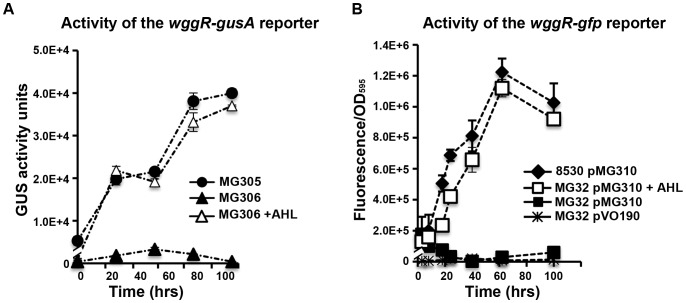
Expression of the *wggR* gene. **A.** Average GUS activity of the *wggR-gusA* merodiploid reporter in the wild type background (MG305, filled circles), *sinI* (MG306 (filled triangles) or in MG306 with 150 nM of C_16:1_-HSL (open triangles) in bacteria grown on surfaces of the soft agar. Averages of three biological replications within a representative experiment are shown, error bars are standard deviation. **B.** Average GFP activity [fluorescence/OD_595_] of *wggR-gfp* fusion reporter plasmid pMG310 in Rm8530 (filled diamonds), in MG32 (filled squares, short dashes), and in MG32 with 150 nM of C16:1-HSL (open squared). Background fluorescence of the pVO190 vector in MG32 is shown as a dashed line with stars.

Under similar conditions, the expression of *wggR* followed a similar time course as *sinI* as indicated by both the chromosomal *wggR*-*gusA* reporters (MG305 and MG306, Fig. 6A) and by the plasmid-borne *wggR-gfp* reporter (pMG310, Fig. 6B). The expression of *wggR* was induced by C_16:1_-HSL (Fig. 6). These results indicate that the expression of *wggR* depends on SinI AHL within spreading colonies, therefore providing important information on the role of the QS in controlling EPS II biosynthesis and its role in swarming. These results are also in an agreement with earlier transcriptional studies [24], [33].

### Specific SinI AHLs restore swarming phenotypes of the *sinI* and the *sinR* mutants, but not the *expR* mutant and EPS II defective mutants

**Figure 7:**
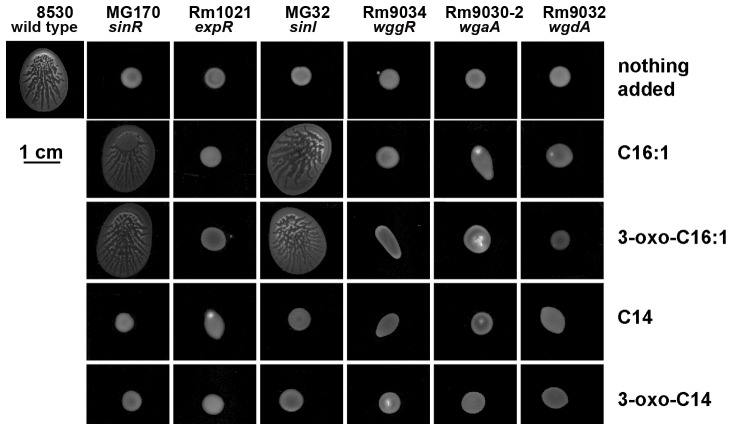
Specific AHLs induce swarming colony formation. Colony spread of *S. meliloti* Rm8530 (wild type), MG32 (*sinI*), MG170 (*sinR*), Rm1021 (*expR*), Rm9034 (*wggR*), Rm9030-2 (*wgaA*), and Rm9032 (*wgdA*) on 0.4% agar containng 1/20 LB medium (top row). Colonies of MG32, MG170, Rm1021, *wggR*, *wgaA*, *wgdA* on the medium containing 200 nM of synthetic C_16:1_-HSL (second row), 3-oxo-C_16:1_-HSL (third row), C_14_-HSL (fourth row) and 3-oxo-C_14_-HSL (bottom row). Inoculated plates were incubated at 30°C for 3 days before being photographed. Genotypes of the strains are on the top of the figure; strain names are on the bottom of the figure.

SinI is known to catalyze the synthesis of at least seven different AHLs [21], [22]. We tested four of SinI AHLs for their ability to facilitate swarming. The addition of 200 nM of C_16:1_- and 3-oxo-C_16:1_ HSLs separately added into the soft agar growth medium restored swarm of the *sinI* mutant MG32 and the *sinR* mutant MG170, but not the *expR* mutant Rm1021 (Fig. 7, top rows). At the same concentration, neither C_14_- nor 3-oxo-C_14_-HSL affected swarming in the *sinI*, the *sinR* and the *expR* mutants (Fig. 7, two bottom rows). This is consistent with earlier observations of C_16:1_ and oxo-C_16:1_-HSLs restoring surface spreading of the *sinI* mutant [22]. The ability of AHLs to restore swarming in the *sinR* mutant is also consistent with the ability of plasmid-borne *sinI* to partially rescue swarming in the *sinR* mutant (Fig. 4A). This further supports the hypothesis that the major function of SinR in *S. meliloti* Rm8530 swarming is to stimulate the synthesis of AHLs by controlling the expression of *sinI* gene.

The addition of other AHLs, including C_8_ -HSL (at 22 µM), C_12_-HSL (18 µM), and C_16_-HSL (3.5 µM), separately added into soft agar growth medium did not restore the swarming phenotypes of the *sinI*, the *sinR*, and the *expR* mutants (data not shown). These results indicate that two specific SinI AHLs (C_16:1_- and oxo-C_16:1_-HSLs) are involved in swarming in ExpR-dependent fashion.

ExpR has a known function of controlling EPS II production. Stimulated by ExpR (and in concert with it), WggR protein interacts with the promoter regions of the operons involved in EPS II biosynthesis and secretion, including *wga* and *wgd* [35]. By testing the swarming phenotypes of the EPS II mutants in the presence or absence of AHLs, we invastigated (1) whether AHLs, as signals, affect swarming indirectly via the ExpR-WggR-mediated EPS II synthesis or (2) whether AHLs function directly as surfactants or surface wetting agents. As shown in Fig. 7, swarming in *wggR*, *wgaA* and *wgdA* mutants was abolished. Neither the addition of 200 nM of C_16:1_-, 3-oxo-C_16:1_-, C_14_-, 3-oxo-C_14_-HSLs (Fig. 7), nor the addition of shorter chain AHLs (C_8_-, C_12_-HSLs at 18–22 µM) (data not shown) restored the ability of these EPS II mutants to swarm over the soft agar surface. These indicate that AHLs function as signals rather than surfactants in Rm8530 swarming motility, and that their regulatory effects on swarming require ExpR- and WggR-mediated regulatory cascades leading to EPS II biosynthesis.

### The effects of *sinI*, *sinR* and *expR* on *sinI* expression

**Figure 8:**
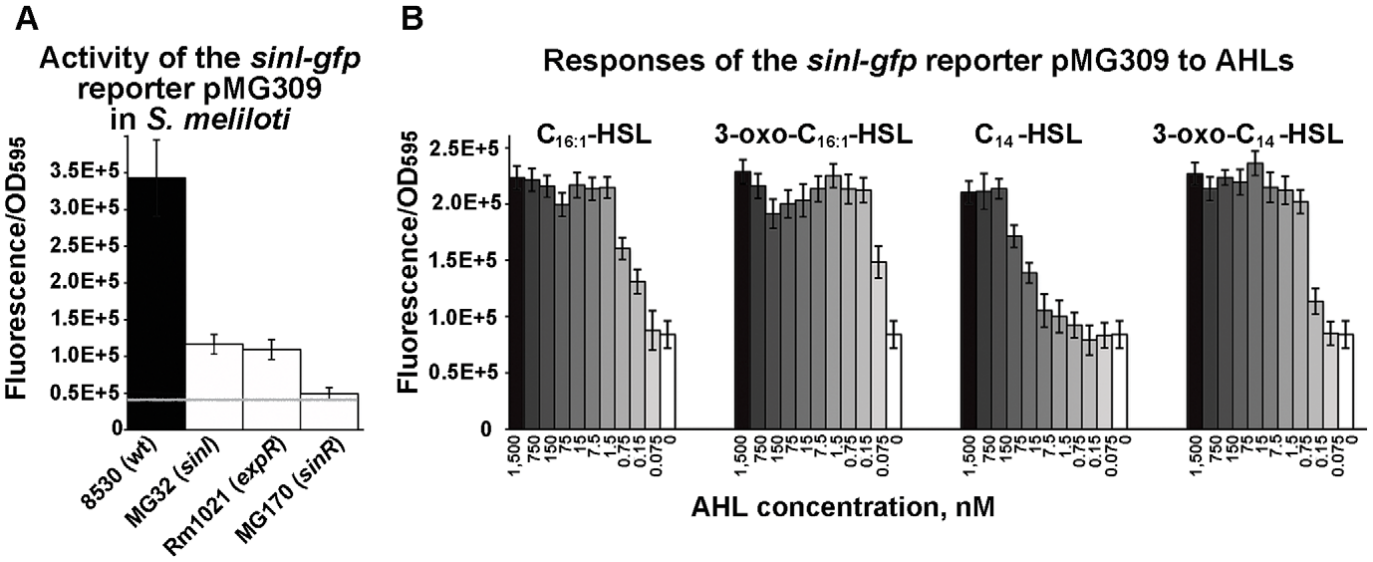
Responses of the *sinI* reporter to AHL signals. **A.** Activity of the *sinI-gfp* reporter pMG309 in *S. meliloti* 8530 (wild type), MG32 (*sinI*), Rm1021 (*expR*) and MG170 (*sinR*). A dashed white line indicates average fluorescence/OD_595_ (41717±2380 units) from Rm8530 and mutants carrying pVO190 vector. **B.** Average activity of pMG309 in the *sinI* mutant MG32 grown on 0.4% agar and 20-fold-diluted LB medium plates containing C_16:1_-, 3-oxo-C_16:1_-, C_14_- and 3-oxo-C_14_-HSLs at the indicated concentrations (or equivalent amounts of the methanol solvent). Bacteria were collected from the spreading colonies that formed after two days of incubation on soft agar. Averages from three technical replications within a representative experiment are shown, error bars are standard deviations. The experiment was repeated twice with reproducible results.

Because QS genes *sinI*, *sinR* and *expR* are needed for swarming to occur, we tested whether or not they contribute to it independently or whether they are all part of one regulatory hierarchy. To address this question, the activity of pMG309 (the plasmid carrying the *sinI-gfp* reporter) was tested in colonies formed by the wild type strain Rm8530 and its isogenic *sinI* mutant MG32, *sinR* mutant MG170 and the *expR* mutant Rm1021 after a two-day incubation on 0.4% agar (Fig. 8A). The disruption of either *expR* or *sinI* reduced the expression of *sinI* by approximately 3-fold, while the deletion of *sinR* had the most severe effect. This is consistent with the observations in liquid media [44]. These observations also match with the phenotypes of the corresponding mutants: *sinR* mutants are unable to produce SinI AHLs, while *expR*-defective strain *S. meliloti* 1021 produces AHLs, although in lower quantities [21], [22].

### The effect of a broad range of AHLs on *sinI* gene expression

Because specific C_16:1_- and oxo-C_16:1_-AHLs restored swarming of the *sinI* and *sinR* mutants (Fig. 7), we tested whether or not this is due to the two specific AHLs stimulated expression of *sinI* gene in bacteira on the soft agar. Fluorescence of pMG309 in the *sinI* background was measured after 2 days of incubation on agar containing different AHLs (Fig. 8B). C_14_-, 3-oxo-C_14_- and C_16:1_- HSLs induced *sinI* gene expression when supplied at 0.15–15 nM and higher concentrations; 3-oxo-C_16:1_ was active at 0.075–0.15 nM and higher concentrations (Fig.8B). C_8_- (at 22 µM), C_12_- (at 18 µM), and C_16_-HSL (at 3.5 µM) induced the *sinI* reporter activity by approximately two fold. Thus, *sinI* appears to respond to a broad range of AHLs with 3-oxo-C_16:1_-HSL being active at the lowest concentrations.

### Specific SinI AHLs stimulate the expression of *wggR*

**Figure 9:**
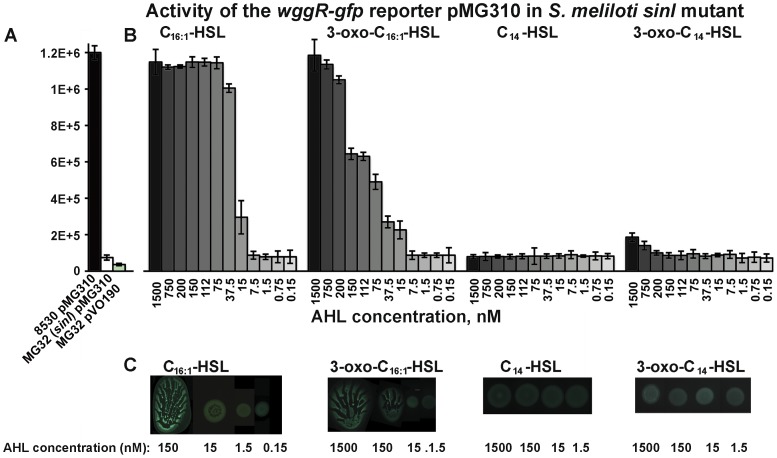
Responses of *wggR* to AHLs. **A.** Average activity [fluorescence/OD_595_] of the *wggR-gfp* reporter pMG310 in *S. meliloti* Rm8530 (wild type), MG32 (*sinI*) and MG32 pVO190 (vector control). **B.** Average activity of *wggR-gfp* (on pMG310) in MG32 grown on 0.4% agar and 20-fold-diluted LB medium plates with dilutions of AHLs (from left to right) C_16:1_-, 3-oxo-C_16:1_-, C_14_-, and oxo-C_14_-HSL. Fluorescence of the MG32 pMG310 reporter in the negative control (solvent only) was 7,995±646. For the assays, bacteria were collected from colonies after 3 days of incubation on soft agar plates. Average of three biological replicas within a representative experiment are shown. All essays were repeated at least twice with reproducible results. Error bars denote standard deviations. **C.** Fluorescent images of colonies formed by MG32 pMG310 on soft agar containing different kinds and amounts of AHLs (as indicated at the bottom of the image).

To address the question of whether specific SinI AHLs stimulate the expression of the *wggR* gene, we first measured and compared the activity of the plasmid pMG310 (carrying the *wggR-gfp* reporter) in the wild type and the *sinI* mutant grown on soft agar. The activity of the *wggR*-*gfp* reporter in the *sinI* mutant was more than 10 times lower than that in the wild type (Fig. 9A). Next, responses of the *wggR-gfp* reporter to different AHLs were measured in the *sinI* mutant. As shown in Fig. 9B, the *wggR-gfp* reporter was unresponsive to C_14_-HSL and only responded weakly to 3-oxo-C_14_-HSL at the two highest concentrations (750 nM and 1500 nM). The activity of the *wggR*-*gfp* reporter increased strongly upon the addition of C_16:1_-HSL and 3-oxo-C_16:1_-HSL, and it did so in a dose-dependent fashion (Fig. 9B). The amount of C_16:1_-HSL that elicited full responsiveness of the reporter was approximately 5-fold lower than that of 3-oxo-C _16:1_-HSL (Fig. 9B). Neither C_14_-, nor 3-oxo-C_14_-HSL restored the swarming phenotype of the *sinI* mutant strain (Fig. 9C). The C_16:1_-HSL and oxo-C_16:1_-HSL stimulated the activity of *wggR-gfp* reporter in the *sinI* mutant correlated with the extent and the appearance of swarming (Fig. 9C). C_8_- (at 22 µM), C_12_-HSL (at 18 µM), and C_16_-HSL (at 3.5 µM) did not induce the *wggR-gfp* reporter. These data strongly suggest that specific C_16:1_- and oxo-C_16:1_- AHL signals stimulates the expression of *wggR* gene in the *sinI* mutant to enhance the regulation of EPS II production that promoted the surface movement.

These observations are consistent with the data shown in Fig. 7 and further suggest that even though the *sinI* promoter is responsive to a fairly broad range of AHLs (Fig. 8B), the synthesis of EPS II is tightly regulated by WggR, and the WggR regulation on EPS II production is tightly controlled by the two specific SinI AHLs C_16:1_- and oxo-C_16:1_-HSL through WggR.

### Crude EPS II and its HMW fractions partially rescue spreading phenotypes of AHL- and EPS II- defective mutants

**Figure 10:**
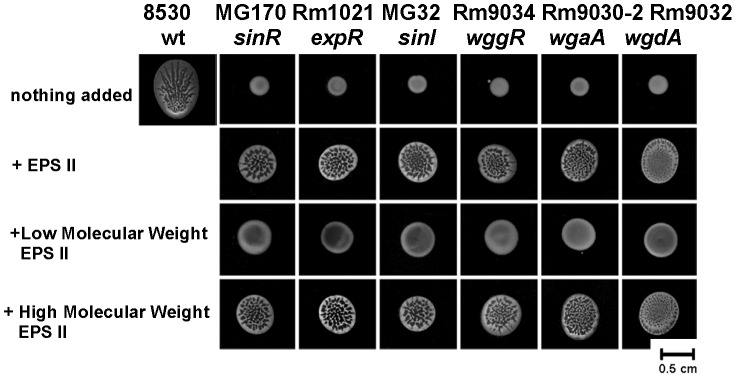
Contributions of EPS II to colony spreading. Surface spreading of *S. meliloti* Rm8530 (wild type), MG32 (*sinI*), MG170 (*sinR*), Rm1021 (*expR*), Rm9034 (*wggR*), Rm9030-2 (*wgaA*), and Rm9032 (*wgdA*) on 0.4% agar and 20-fold-diluted LB medium (top row). Crude EPS (harvested from the spreading colonies of Rm8530) or its high or low molecular weight fractions (20 µl) were spotted onto the plate surfaces, into which 3.5 µl of tested bacteria were added. The amount of supplied EPS II represents ∼1/2–1/7 of the amount of EPS II that is produced by a 3-day old single swarming colony. Plates were incubated for 2–3 days at 30°C before photographing.

To test whether EPS II is responsible for facilitating the bacterial spreading over the soft surfaces, EPS II was collected from swarming colonies formed by wild type strain Rm8530, centrifuged, filter-sterilized, and then size-fractioned. Crude extracellular matrix harvested from swarming colonies formed by wild type partially restored the swarming defect phenotypes of the EPS II mutants Rm9034 (*wggR*), Rm9030-2 (*wgaA*), and Rm9032 (*wgdA*) to the stage I of swarm (Fig. 10, see also Fig. 1 for the morphology of a normal swarming colony at stage 1). The same phenotypes were observed for the QS mutants MG170 (*sinR*), Rm1021 (*expR*) and MG32 (*sinI*) suggesting that their contributions to swarming involve controlling the production of EPS II (Fig. 9). Crude EPS isolated from the 2–3 day old colonies of the *wgaA* mutant grown on soft agar did not restore the swarming of any mutants tested in Fig. 10 (data not shown), indicating that other surface polysaccharides do not play a role in this type of swarming. This is expected, because none of EPS II mutants was able to swarm as shown in Fig. 10 and the EPS I mutant formed normal swarming colonies (Fig. 3).

Because EPS II produced by Rm8530 is known to exist as LMW and HMW fractions, we size-fractionated the collected extracellular matrix (EPS II) to test which component had the greatest effect on swarming. As shown in Fig. 10, the LMW fraction had no effect on swarming. The HMW fraction partially restored the swarming in the mutants defective in the AHL synthesis and perception (*sinI, sinR and expR*), and in the mutants defective in EPS II synthesis (*wggR, wgaA, wgdA*). The extent of complementation by the HMW fraction was the same as with the crude EPS II. This suggests that the HMW fraction of EPS II produced by *S. meliloti* in the ExpR-dependent manner contributes to the early stages of swarming. This is the first reported function for HMW EPS II.

### Expression of *wggR* in subpopulations of Rm8530 swarming cells

The microscope imaging (Fig. 1) showed patterns of uneven distribution of population inside Rm8530 swarming colonies and suggests that production or uptake of AHL signals by individual cells may occur differently under those conditions. Therefore, we investigated whether or not *wggR* gene was differentially expressed among individual cells under similar conditions. For this purpose, a resolvase-based *in vivo* expression technology (RIVET) [46] was used to detect *wggR* promoter activity of individual cells within colonies on soft agar.

The RIVET method [46] is based on the ability of TnpR recombinase to catalyze site-specific “resolution” at *res* sequences. When *tnpR* is expressed from a promoter of interest, activation of this promoter drives the expression of the recombinase gene. TnpR then excises a selectable tetracycline resistance gene. This resolution event causes the loss of the tetracycline marker and generates tetracycline sensitive progeny. Therefore, the resolution of RIVET reporter bacterial cells, calculated as percentage of the tetracycline-sensitive colonies over the total, are used to indirectly measure the activity of the promoter that drives the expression of *tnpR* [46]. *S. meliloti* MG102 [47] is a *wggR* RIVET reporter of Rm8530 strain. It harbors a chromosomal integrated *wggR-tnpR* resolvase gene fusion and a *res-tet-res* cassette inserted in a neutral site of Rm8530 chromosome [47]. Similarly, MG103 is a *wggR* RIVET reporter of the *sinI* mutant strain. MG103 reporter was constructed by introducing the *wggR-tnpR* gene fusion and the *res-tet-res* cassette into the chromosome of the *sinI* mutant using the protocol previously described [47]

Average resolution of MG102 was 28±3% in triplicate swarming colonies at its third day of swarming on soft agar. The average resolution of MG103 reporter was low in non-swarming colonies under similar conditions. A 3-day-long growth of MG103 on soft agar containing 15 and 150 nM of C_16:1_-HSL stimulated 10±5% and 30±3% resolutions of the MG103 reporter, respectively, compared with 3.3±1.5% resolution of the reporter grown on soft agar without C_16:1_-HSL.

These results indicate that approximately 1/3 individual cells in the swarming colonies increased the expression of their *wggR* gene in response to C_16:1_-HSL signals and the rest of them did not. Thus, it appears that SinI C_16:1_-HSL enhanced the expression of the *wggR* in subpopulations to upregulate their EPSII production, and this was sufficient to support a population-wide swarming. How did cells in which the expression of *wggR* remained at low levels contribute to swarm of Rm8530 remain unknown.

### Spreading of colonies in the presence of “cheater” mutants

**Figure 11:**
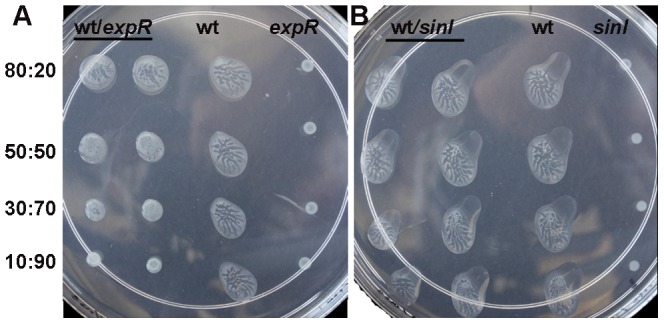
Co-spreading of *S. meliloti* 8530 and mutants. Swarming colonies formed by the mixture of Rm8530 (wild type) and Rm1021 (*expR*) (left), or by the mixture of Rm8530 and MG32 (*sinI*) (right). Surface of soft agar were inoculated with mixed inocula contain a mutant and 80%, 50%, 30% and 10% of Rm8530. As a control, pure cultures of Rm8530 and mutants were spotted separately on the same plate. Photos were taken after two days inoculation.

The discovery that only a subpopulation of the cells within a colony contributed to the production of the extracellular EPS II in the QS-dependent manner raises important questions about the population-wide effects of QS, resource allocations within bacterial communities, and the role of “cheaters” [17]. For the co-spreading assays, EPS II and QS mutants were used. As shown in Fig. 11A, the average diameter of co-spreading colonies formed by mixing the wild type and the *expR* mutant (8∶2) was 15% less than that of the wild type. Diameters of the co-spreading colonies were further reduced with the increased proportion of the *expR* mutant (Fig. 11A). Similar results were obtained with the spreading co-cultures of the *wggR* and *wgaA* mutants (data not shown). Mixed spreading colonies formed by the *sinI* mutant MG32 and Rm8530 were identical to those of the wild type colonies even when the mutant made up the majority of the population (Fig. 11B). These results are reminiscent of those obtained in *P. aeruginosa* where the “public goods” cheaters were more detrimental to the colony than “signal cheaters” [17].

## Discussion

### Swarming behavior

Soto et al. [7] first observed surface swarming in *S. meliloti*. Their G4 WT strain did not swarm under the conditions tested, but a *fadD* mutant did. Our results show that *S. meliloti* Rm8530 strain can swarm on very soft agar (0.4%). The *fad* mutant swarming cells were hyperflagellated, and they stopped their propagation in swarming colonies [7]. Rm8530 swarming cells were not hyperflagellated (Figure 2A), and they did not stop their propagation in swarming colonies (Figure. S1). Social and cooperative behaviors are known to occur in swarming colonies in other bacteria [14], [15], and the swarming of Rm8530 was dependent on controlled secretion of EPS II, consistent with involvement of social organization in swarming colonies. Dual motility systems (A-motility and S-motility) in soil bacterium *Myxococcus xanthus* were reported [48], and those motilities show different selective advantages on various surfaces [48]. Swarming of Rm8530 studied here is one of a few motility phenomena described in *S. meliloti* so far [7], [9], [27], and likely to help the bacteria to adapt complex surface environments.

### Regulation of swarming behavior

The need for ExpR/Sin QS system to initiate swarming colony in *S. meliloti* Rm8530 seems to be restricted to generating AHL signals perceiving the AHL signals, and regulating the EPS II production (Fig. 4 and Fig. 7). The ability of SinI C_16:1_-HSL and oxo-C_16:1_-HSL to stimulate swarming of the *sinI* mutant and the *sinR* mutant but not the *expR* mutant, indicates that swarming colony initiation on the soft surface involves these specific SinI AHLs acting as signals mediated by the ExpR receptor. The inability of C_16:1_-HSL and oxo-C_16:1_-HSL to stimulate swarming of the *wggR* mutant indicates that these AHLs contribute little to swarm in the absence of WggR. Thus, it appears that *sinI* made C_16:1_-HSL and 3-oxo-C_16:1_-HSL activate the ExpR receptor and this directly or indirectly enhances expression of *wggR* and contributes to the regulation of the production of EPSII. This relationship is consistent with earlier transcriptional studies [24], [26], [27], [33]. In addition to enhancing the expression of *wggR*, C_16:1_-HSL was shown to restore the expression of other EPS II genes at the presence of ExpR [26]. Current data show that positive regulation of EPS II genes by ExpR is dependent on WggR [27]. These explain why an overexpressed *wggR* is incapable to stimulate swarming in the *sinI* mutant (Figure 4B).

The inability of C_14_–HSL and oxo-C_14:1_–HSL to stimulate swarming of the *sinI*, the *sinR*, the *expR*, or the *wggR* mutants (Figure 7) suggests that those SinI AHLs normally do not act as signals for the initiation of Rm8530 swarming. The inability of C_14_ and oxo-C_14_- AHL to stimulate *wggR* promoter in the presence of ExpR (Fig. 9B) strongly supports our finding that the expression of *wggR* is specifically stimulated by the C_16:1_- and oxo-C_16:1_-HSLs activated ExpR. Interestingly, earlier gel shift assays showed that oxo-C_14:1_–HSL did not enhance the relationship between ExpR and the *wggR* promoter [27]. The inability of overexpressed *sinI* and *sinR* genes (Figure 4B) and synthetic AHLs (Figure 7) to stimulate swarming of EPS II mutants indicate that SinI AHLs do not normally act as surfactants and/or wetting agents in Rm8530 swarming cells.

The levels of *sinI* expression in a *sinI expR* double mutant were not significantly affected by the addition of C_16:1_-HSL or oxo-C_16:1_-HSL added into soft agar (Figure S2B). These results support our conclusion that the initiation of swarming depends on the interaction of these AHLs with ExpR. The levels of *sinI* expression in the *sinI expR* double mutant were significantly increased by the addition of other AHLs (oxo-C_14_-HSL, C_12_-HSL and C_8_-HSL) (Fig. S2A), raising the question of whether or not these signal molecules interact with the SinR protein or other predicted LuxR-like proteins to affect the expression of *sinI*. The answer to that question remains unknown.

EPSII is secreted in two major fractions: HMW and LMW. We have shown that the HMW fraction facilitated the initial stages of swarming and that LMW fraction is not critical for facilitating the initial stages of swarming (Figure 10).

This study demonstrates that swarming is a social behavior that can be encouraged or discouraged by changes in QS signaling input and the regulation in gene expression. While the influence of QS on swarming is studied in the aspect of *wggR*, other regulatory gene products may also contribute to the behavior through their effects on the production of EPS II and motility genes. For example, MucR, a RosR homolog, is a positive regulator of EPS I gene and a negative regulator of EPS II genes including *wggR* [35]. MucR mutants produce HMW EPS II exclusively [30]. ExpR/Sin QS system increases expression of the *wggR*. WggR derepresses EPS II production at the transcriptional level from MucR, while concurrently elevating the expression of *wgeA*, resulting in the synthesis of the LMW fraction [33]. The role of MucR in controlling swarming of Rm8530 remains to be investigated.

## Materials and Methods

### Media and culture conditions

For routine propagation, strains of *S. meliloti* were grown at 30°C in TY broth [49]. *E. coli* was cultured in Luria-Bertani broth (Fisher Scientific, Fair Lawn, New Jersey 07410, U.S.A.) at 37°C. As needed, media were supplemented with antibiotics at the following final concentrations: streptomycin, 250–500 µg/ml; neomycin, 100 µg/ml; tetracycline, 2.5–5 µg/ml; gentamicin, 50 µg/ml; kanamycin, 25 µg/ml. C_16:1_-Δ^9^ *cis*-(L)-homoserine lactone (referred to as “C_16:1_-HSL” in text), 3-oxo-C_16:1_-Δ^11^ *cis*-(L)-homoserine lactone (referred to as “3-oxo-C_16:1_-HSL” in text), 3-oxo-C_14_- and C_14_-homoserine lactones were from Cayman Chemical (Ellsworth Road, Ann Arbor, MI, U.S.A.). Other AHLs were from Sigma-Aldrich (St. Louis, MO, U.S.A.).

### Strains and plasmids

Strains and plasmids used in this study are listed in Table 1. Primers are listed in Table 2. For complementation and epistasis studies, the intact ORFs including predicted regulatory regions were PCR amplified from genomes of *S. meliloti* Rm8530 or Rm1021 with the following primers: for p*sinI*, primers MG460 and MG461; for pKB*expR*, MT10 and MT11; for pKY*wggR*, DC3 and DC10; for p*sinR*, MG1866 and MG18677. The amplified fragments were initially cloned into pCR2.1 (Invitrogen, Carlsbad, CA, U.S.A.), and then sequenced. Once confirmed, fragments were released from pCR2.1 with the following restriction endonucleases: *EcoR*I (for the *sinI*, the *expR*, and the *sinR* fragments), *Spe*I, *Xba*I (for the *wggR* fragmnet) and then cloned into pBBRMCS1-based vectors. [50]. Plasmid p*expR*-km was constructed by releasing the *EcoR*I fragment from pKB*expR* and inserting it into *EcoR*I site of pBBR1-MCS2 (Km). Final constructs were confirmed by diagnostic restriction digests and sequencing. Validated constructs were mobilized from *E. coli* into rhizobia by tri-parental conjugation as previouly described [51].

**Table 1:** Strains and plasmids used in this study.

Strain or plasmid	Relevant characteristic(s)	Reference
**Plasmids**		
pBBR1MCS	Broad-host-range cloning vectors	[56]
pDG71	Constitutive P*trp*-Gfpmut3, Tc	[38]
pJQ200SK	*sacB* suicide vector, Gm	[50]
pRK600	Conjugal transfer helper plasmid, Cm	[57]
pTH113	pRK7813, has an intact *sinR and sinI*, Tc	[45]
pVMG	pUC119 derivative, promoterless *gus* with upstream stop codons, Nm	[22]
pVMG*sinI7*	pVMG derivative, *sinI-gus*, Nm	[47]
pVO3	pVMG derivative, *wggR-gus*, Nm	[47]
pVO3TnpR	pVO3 derivative, *wggR-tnpR-gus*, Nm	[47]
pVO190	pBBR derivative containing a promoterless gfpmut1, Sp	Oke,Valerie
pKB*expR*	pBBR1MC-S5 containing a 1.3-kb *Eco*R1 fragment of *expR*, Gm	This work
pKY*wggR*	pBBR1MC-S3 containing a 1.1-kb *Spe*I-*Xba*I fragment of *wggR*, Tc	This work
pMG307	pJQ200SK containing a 1.9-kb *Spe*I-*Apa*I fragmet of *sinR* mutant, *Avr*II, Gm	This work
pMG309	pVO190 containing a 348-bp *Kpn*I-*Xho*I fragment of *sinI* promoter region	This work
pMG310	pVO190 containing a 764-bp *Kpn*I-*Xho*I fragment of *wggR* promoter region	This work
p*sinI*	pBBR1MC-S5 containing a 2.2-kb *Eco*RI fragment of *sinI*, Gm	This work
p*sinR*	pBBR1MC-S5 containing a 1.9-kb *Eco*RI fragment of *sinR*, Gm	This work
***S. meliloti***		
Rm1021	SU47, *expR*102::IS*Rm*2011-1, Sm	[58]
Rm8530	Rm1021, *expR+* Sm	[24]
Rm9030-2	Rm8530, *wgaA* (*expA1*)::*lacZ*-Gm, Sm, Gm	[24]
Rm9032	Rm8530, *wgdA* (*expD1*)::*lacZ*-Gm, Sm, Gm	[24]
Rm9034	Rm8530, *wggR* (*expG*)::lacZ-Gm, Sm, Gm	[24]
Rm11601	Rm8530, *flaA flaB*, Sm, Hy	[40]
Rm11603	Rm8530, *exoY*, Sm,	[6]
RmG910	Rm1021, *fliP*::kan, Sm, Km	[41]
MG32	Rm8530, Δ*sinI*, Sm	[22]
MG32rtr	MG32, with integrated *resI-tet-resI* cassette, Sm, Tc	[47]
MG75	Rm1021, Δ*sinI*, Sm	[22]
MG102	Rm8530 *wggR+*, *wggR-tnpR*, *res1-tet-resI*, Sm, Nm, Tc	[47]
MG103	MG32rtr, with integrated pVO3TnpR, Sm, Nm, Tc	[47]
MG170	Rm8530, Δ*sinR*, Sm	This work
MG301	Rm8530, with integrated pVMGsinI7, *sinI-gusA*, Sm, Nm	This work
MG302	MG32, with integrated pVMGsinI7, *sinI-gusA*, Sm, Nm	This work
MG305	Rm8530, with integrated pVO3, *wggR-gusA*, Sm, Nm	This work
MG306	MG32, with integrated pVO3, *wggR-gusA*, Sm, Nm	This work
MG320	Rm8530, *fliP*::kan, Sm, Km	This work

GUS: ß-glucuronidase; Sm, Sp, Km, Nm, Tc, Gm, Hy: resistant to spectinomycin, streptomycin, kanamycin, neomycin, gentamicin, hygromycin respectively.

**Table 2:** Primers used for cloning.

Primer Name	Sequence	Purpose
DC3	TTGGGGCCCTTGCTAATCAAAGGA	Construction of pKY*wggR*
DC10	ACGAATGCTACATGCATC	Construction of pKY*wggR*
MG43	ggggtACCGGGCCGGAAACGGAGG	Construction of pMG309
MG44	ccgctcgAgTTTTTCGCTCCATGCG	Construction of pMG309
MG45	ggggtaccACGACGGAGATCGC	Construction of pMG310
MG46	ccgctcgAgTGGGAACGTACTTCCAA	Construction of pMG310
MG460	GAAGAAATCGGGCTTTCCACCGA	Construction of p*sinI*
MG461	CGTCGCGAGCACATGATAGTAGAG	Construction of p*sinI*
MG497	ACGATCGTGCGCACGAATACGA	Construction of pMG307
MG1751	TAGATTTCGGCGGcCTaGGCGCCGAAAGT	Construction of pMG307
MG496	ACATCGGGCGATCGAGAACGG	Construction of pMG307
MG1752	ATATCctAGgGAACGGTGCGTTTCTT	Construction of pMG307
MG1866	ACGATCGTGCGCACGAATtCGA	Construction of p*sinR*
MG1867	TGCGACCGgaTCCGTTCACTAT	Construction of p*sinR*
MT10	TTTGCGTTCTTCCCAAAAAACGCGGTA	Construction of pKB*expR*
MT11	AA TGAAGCGCAATTTCAGATGCGACAT	Construction of pKB*expR*

Lowercase letters in oligonucleotide sequences indicate nucleotides that were modified from the published sequence to create enzyme sites.

Plasmid-borne promoter-*gfp* reporter fusions were constructed by PCR amplifying a genomic region containing desired promoters with the following primers: for pMG309 (*sinI-gfp*), MG43 and MG44; for pMG310 (*wggR-gfp*), MG45 and MG46. The PCR fragments were cloned into *Kpn*I/*Xho*I sites in front of the promoterless *gfp* gene in the broad-host-range pVO190 plasmid (a gift from Dr. Valerie Oke). Resulting plasmids, after sequencing, were introduced into *S. meliloti* by tri-parental conjugations as described [51].

To make chromosomal transcriptional fusions, chromosomal integrative plasmids carrying *S. meliloti* DNA were introduced into proper *S. meliloti* strains as described [22]. Plasmid pVO3TnpR was used to make *S. meliloti wggR* RIVET reporter strain MG103; pVMG*sinI*7 was used to make *S. meliloti sinI-gusA* reporter strains MG301 and MG302; pVO3 was used to make *S. meliloti wggR-gusA* reporter strains MG305 and MG306.

MG320 mutant (Rm8530 *fliP*) was created by transducing the *fliP*::Km mutation from RmG910 [41] into Rm8530 using ØM12 as previously described [41]. The mutant isolate was backcrossed one time to Rm1021. Swimming motility defects of MG320 were confirmed by the swimming assay using an established protocol [52].

### Construction of *S. meliloti* MG170 (Δ*sinR*)

To create the *sinR* deletion mutant MG170, two DNA regions flanking *sinR* gene were PCR amplified from the genomic DNA of *S. meliloti* Rm1021 using primer pairs MG497 and MG1751; and MG496 and MG1752, respectively. The two PCR fragments were digested with *AvrII*, purified, ligated to each other. This created a 1.9-kb fragment of *sinR* deletion mutant (the deletion was from 200 to 670 nt within *sinR* open reading frame). The fragment of *sinR* mutant was PCR amplified using primers MG497 and MG496, cloned into pCR2.1 (Invitrogen) and confirmed by sequencing. The fragment of confirmed *sinR* mutant was excised as a 1.9-kb *Spe*I/*Apa*I fragment and cloned into the suicide vector pJQ200SK [50], yielding pMG307. pMG307 was mated into *S. meliloti* Rm8530 with helper plasmid pRK600 [53]. Gentamicin resistant transconjugants containing a single crossover in *sinR* was selected. Sucrose counterselection for double recombinants was performed using 5% sucrose as previously described [22], [50]. Both the presence of the *sinR* deletion and the absence of wild type *sinR* in MG170 mutant was confirmed by PCR and sequencing. The defect in the AHL production of MG170 was confirmed by a bioassay-coupled thin-layer chromatography (TLC) method using an established protocal [22].

### Swarm conditions and the collection of extracellular polymers

#### Swarm conditions

Log-phase cultures (OD_600_ = 0.4 to 0.8) of bacteria, grown in TY broth (30°C, 225 rpm) were centrifuged and the pellets were re-suspended in the original volume in sterile water. Typically, 3.5 µl of bacterial suspensions were spotted onto the surface of 1/20 LB solidified with 0.4% Molecular Genetics grade agar (Fisher Scientific). Prior to the inoculation, plates were cooled for 10 min with lid open on a sterile flow bench. AHLs were added to agar plates. Plates were incubated at 30°C in an upright position, and the appearance of colonies was observed daily.

#### EPS collection

Extracellular matrix was collected from the edges of the spreading colonies in *S. meliloti* 8530 using blunt-ended pipette (∼100–150 µl per colony) and transferred into eppendorf tubes, spun at top speed and then the supernatant was spin-filtered using centrifugal nylon filters (pore size 0.22 µm) to remove bacteria. The bacteria-free preparation was further fractionized into HMW EPS and LMW EPS with a Millipore Ultra Free MC 30,000 NMWL Filter Units (Millipore Corporation, Bedford, MA) following the manufacturer's protocol. Aliquots of the material were routinely checked for the absence of bacteria by plating onto TY agar. EPS from the EPS II mutant Rm9030-2 (*wgaA*) was collected from a plate with 40 dry colonies formed by Rm9030-2 bacteria on surface after 2 days of incubation. This was done by flooding the plate with 2 ml of sterile water, letting the plate stand for 2 minutes, and using blunt-ended pipette to transfer aqueous suspensions containing EPS from the mutant, and then following the same process described above to prepare bacteria-free EPS. 20 µl of the preparation was used for experiments.

### β-Glucuronidase (GUS) assays

Cells grown on the soft agar were collected from the surfaces at the indicated times by flooding plates with 1.5 ml of water for 1 minute and then carefully aspirating bacterial suspensions using an established protocol [54]. After optical density of the samples was measured at 595 nm (OD_595_), cells were permeabilized with lysozyme (200 µg ml^−1^, 37C for 10 min), and the activity was assayed with p-nitrophenyl-β-*o*-glucuronide. GUS activity is presented in nanomoles per minute per OD_595_ unit ×1,000 as in [55].

### GFP measurement

Cells were collected from agar surfaces using the same way as in the GUS assay and suspended in water. Quantitative green fluorescence was measured as [Fluorescence (1.0 s)(Counts)/OD595] in 96-well microliter plates using Wallac 1420, Multilabel counter with the filter set for fluorescent measurement (485-nm band pass excitation filter and a 535-nmbarrier filter)(PerkinElmer Life and Analytical Science, Wallac Oy, P.O. Box 10, FIN-20101 Turku, Finland).

### Imaging

Pictures of spreading colonies were taken with a gel doc imager or with a Canon EOS Rebel XSI camera. Images were acquired from Olympus MVx10 dissecting scope equipped with a *gfp* filter) with MicroFirs camera (Optitronics, Goleta, CA, USA). Images were then transferred into Adobe Photoshop CS, brightness and color balance were normalized using default automatic settings. Multi-panel images were assembled in Adobe Photoshop CS.

## Supporting Information

Figure S1 **Bacterial growth within spreading colonies.** **A.** Spreading colony formed by *S. meliloti* Rm8530 (wild type) contains more cells than non-swarming colonies formed by *sinI*, *sinR* and *expR* mutants (based on OD_600_ measurements), implying benefits for colony growth. **B.** Colony appearances of wild type and mutant bacteria on agar surface from which cells were harvested.
(TIF)

Figure S2 **Effect of AHLs on** ***sinI*** **expression in a** ***sinI expR*** **double mutant.** **A.** Comparison of C_16_-, C_12_- and C_8_-HSL induced GFP activity of *sinI-gfp* (pMG309) in MG32 (*sinI*) and MG75 (*sinI expR*). **B.** Average GFP activity of the *sinI*-GFP promoter plasmid (pMG309) in MG75 (*sinI expR*) mutant with or without AHLs added into soft agar. Bacteria were from colonies after two days incubation on soft agar. Each data point is an average of three technical replications from a representative experiment. Error bars are standard deviations.
(TIF)
