# Supplementary figures and images for: Role of Specific Quorum-Sensing Signals in the Regulation of Exopolysaccharide II Production within Sinorhizobium meliloti Spreading Colonies
Source: PLoS One. 2012 Aug 13;7(8):e42611. doi: 10.1371/journal.pone.0042611 (PMC3418255; doi:10.1371/journal.pone.0042611)

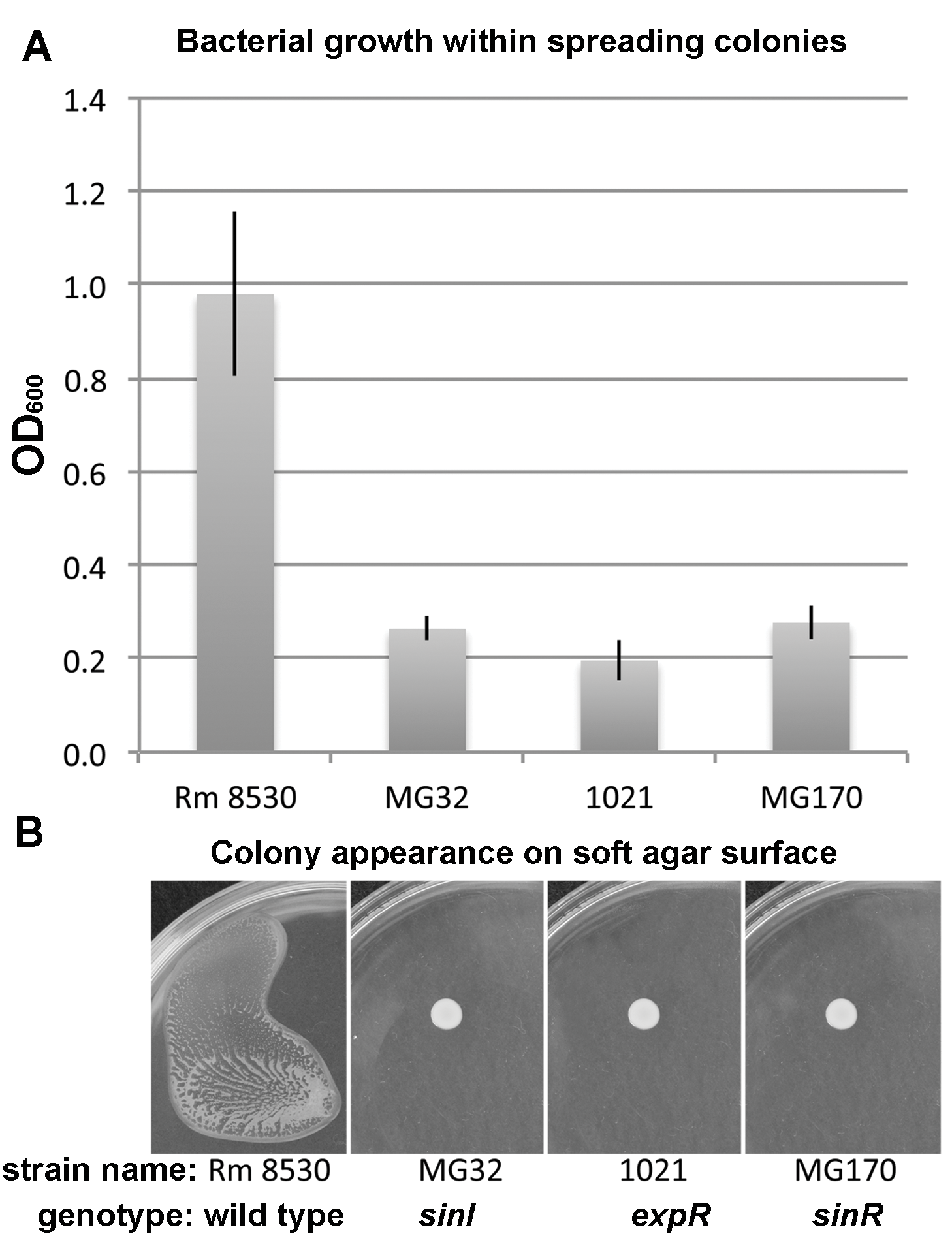

Supplement: Figure S1 — Bacterial growth within spreading colonies. A. Spreading colony formed by S. meliloti Rm8530 (wild type) contains more cells than non-swarming colonies formed by sinI, sinR and expR mutants (based on OD600 measurements), implying benefits for colony growth. B. Colony appearances of wild type and mutant bacteria on agar surface from which cells were harvested. (TIF) [file pone.0042611.s001.tif]

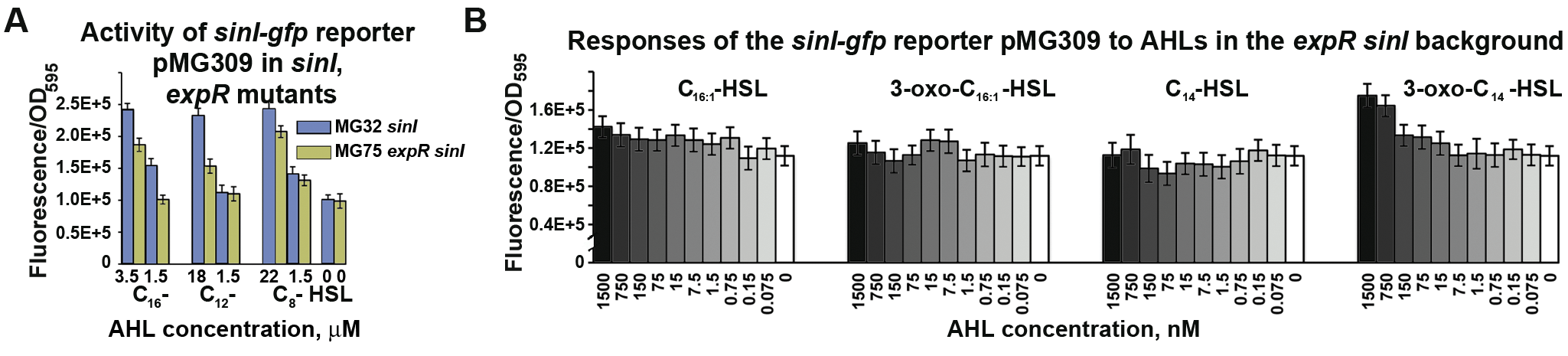

Supplement: Figure S2 — Effect of AHLs on sinI expression in a sinI expR double mutant. A. Comparison of C16-, C12- and C8-HSL induced GFP activity of sinI-gfp (pMG309) in MG32 (sinI) and MG75 (sinI expR). B. Average GFP activity of the sinI-GFP promoter plasmid (pMG309) in MG75 (sinI expR) mutant with or without AHLs added into soft agar. Bacteria were from colonies after two days incubation on soft agar. Each data point is an average of three technical replications from a representative experiment. Error bars are standard deviations. (TIF) [file pone.0042611.s002.tif]
